# Differential effects of reward and punishment in decision making under uncertainty: a computational study

**DOI:** 10.3389/fnins.2014.00030

**Published:** 2014-02-21

**Authors:** Elaine Duffin, Amy R. Bland, Alexandre Schaefer, Marc de Kamps

**Affiliations:** ^1^School of Computing, University of LeedsLeeds, West Yorkshire, UK; ^2^Neuroscience and Psychiatry Unit, University of ManchesterManchester, UK; ^3^School of Business, Monash UniversityBandar Sunway, Malaysia

**Keywords:** decision making, uncertainty, volatility, reward and punishment, Bayesian learning, reinforcement learning

## Abstract

Computational models of learning have proved largely successful in characterizing potential mechanisms which allow humans to make decisions in uncertain and volatile contexts. We report here findings that extend existing knowledge and show that a modified reinforcement learning model, which has separate parameters according to whether the previous trial gave a reward or a punishment, can provide the best fit to human behavior in decision making under uncertainty. More specifically, we examined the fit of our modified reinforcement learning model to human behavioral data in a probabilistic two-alternative decision making task with rule reversals. Our results demonstrate that this model predicted human behavior better than a series of other models based on reinforcement learning or Bayesian reasoning. Unlike the Bayesian models, our modified reinforcement learning model does not include any representation of rule switches. When our task is considered purely as a machine learning task, to gain as many rewards as possible without trying to describe human behavior, the performance of modified reinforcement learning and Bayesian methods is similar. Others have used various computational models to describe human behavior in similar tasks, however, we are not aware of any who have compared Bayesian reasoning with reinforcement learning modified to differentiate rewards and punishments.

## 1. Introduction

Humans need to identify patterns or regularities in their environment in order to survive. Such patterns may be hard to discern due to natural randomness, or noise, in the environment. In addition, underlying patterns may change unexpectedly, making previously learnt behavior inadequate. Yu and Dayan (2005) made the distinction between *expected* and *unexpected uncertainty* in this regard. Situations in which underlying rules change unpredictably at different intervals are often called *volatile* (e.g., Behrens et al., 2007; Bland and Schaefer, 2012). The interaction of these different forms of uncertainty poses a challenge for successful learning, and recent research has tried to understand how humans adapt to such environments (e.g., Hampton et al., 2006; Behrens et al., 2007).

In this paper, we consider computational models which best describe human learning under expected uncertainty in a volatile environment (Bland and Schaefer, 2011). On multiple trials, participants pressed one of two buttons in response to one of two visual stimuli. Participants learnt from being rewarded with points or punished by a deduction of points after each response. Rewards and punishments were generated according to underlying rules which formed a probabilistic Stimulus-Response association, creating expected uncertainty. A volatile environment with frequent unsignalled switches in Stimulus-Response rules was contrasted with stable periods with no such switches. Participants only received win and loss feedback and were not given any explanation about the rules by which stimulus and feedback were generated, nor were they told there were such rules.

Within our computational models, we apply different parameters in response to a win and a loss. Such a differentiation is suggested by the neuroscience and psychology of decision making. Many studies have explored the concept of loss-aversion which states that behavior changes more in response to losses than to gains of similar magnitude (Kahneman and Tversky, 1984). The wide reporting of loss-aversion has prompted the investigation into its neural basis. Tom et al. (2007) used fMRI imaging to investigate responses to theoretical gambles and found brain regions which showed increasing activity for increasing potential gains and the same regions decreased in activity as potential losses increased.

Such studies can give an indication of the brain regions involved in response to wins and losses, but not the actual mechanisms. The neurotransmitter dopamine produced in the midbrain affects two different classes of dopamine receptors in the striatum, which differentially respond to rewards and punishments (see e.g., Gerfen, 1992). More recently, differential neural responses to wins and losses have been found more widely than dopamine receptors in the striatum. Kravitz et al. (2012) found the striatal dopamine receptors to be part of separate neural pathways for reward and punishment. The work of Matsumoto and Hikosaka (2009) suggests that differential responses may occur earlier in processing, they found evidence for different groups of dopamine neurons responding to positive and negative motivation in the midbrain of primates.

Given their widespread use in similar studies, we used two machine learning approaches to model human behavior in the task considered here: Bayesian learning and reinforcement learning (e.g., Hampton et al., 2006; Behrens et al., 2007). Bayesian learning requires prior assumptions about the causal structure of an environment, and when those assumptions are correct, the performance is optimal given only the information available up to a trial. Reinforcement learning does not depend on existing assumptions, but may not be appropriate in changing environments (Sutton and Barto, 1998).

Despite the strong indications in neuroscience and psychology of a differential response to reward and punishment, few studies comparing alternative computational approaches to learning from experience in dynamic environments have considered separate effects of reward and punishment, as noted by Yechiam and Hochman (2013b). Ito and Doya (2009) and Guitart-Masip et al. (2012) are examples of studies which differentiate learning from rewards and punishments by fitting different reward values following a win or a loss rather than using different learning rates. Although these studies compared alternative learning mechanisms, neither considered Bayesian models which required assumptions about the nature of the environment. In a task using probabilities which were known in advance, Charness and Levin (2005) compared a Bayesian learning model to reinforcement learning and found that participants made errors in circumstances when the prediction of the two models differed. They amended the reward structure of the task and found fewer errors, suggesting that the affect resulting from outcomes plays a big part in reinforcement learning.

We use computational learning methods to try to explain our participants' behavior on a trial by trial basis. For our task, we find that a modified reinforcement learning model is better able to account for human behavior than both Bayesian models and standard reinforcement learning, even after taking into consideration the number of free parameters in each model. Our modification to reinforcement learning differentiates learning according to whether the participant won or lost on the previous trial (WL model). Despite significant between-subjects variability in task performance and differences in experimental conditions, we find that each participant's behavior can be described by an individual set of learning parameters which remain constant over all trials. In addition, we find significant differences between the fit values for the parameters after a win and those after a loss. Earlier modeling work on a similar task predicts that learning rates change in response to feedback given (Behrens et al., 2007). In our particular task, we could explain human behavior without variable learning rates.

Our WL model has four parameters: learning rate after a win and loss and temperature after a win and a loss. The temperature allows for variability between participants in the probability of responding in accordance with the current underlying belief. For most participants we find a higher learning rate after a win than a loss, and a higher temperature after a loss than a win. These four parameters can be interpreted as a characterization of the participant's individual strategy. Interestingly, when we consider the task just as a computational challenge without aiming to model human behavior, we find that our WL model is just as good as Bayesian models at the task. The WL model is significantly better at the task than standard reinforcement learning.

We are aware that the experimental paradigm reflects a relatively simple task in terms of real world problems. In many other tasks reinforcement learning strategies will not do so well. A three-choice task would already require more exploration, for example. Nevertheless, the task is interesting as a minimal model of changing rules in a noisy environment. It is clear that after differentiating between a win and a loss, reinforcement learning strategies do very well on our task, and a further exploration of similar tasks would be very interesting.

## 2. Results

### 2.1. Human behavior

In this task, participants were repeatedly shown a series of colored triangles, red or blue, on a screen, and they were required to press one of two buttons in response to each triangle. They received monetary reward or punishment according to whether this response was correct or wrong. To analyze the behavior, we encoded each response according to underlying behavioral types, where type 1 behavior was to press button one when a red triangle was shown, and button two for blue; type 2 was the opposite of type 1. Over groups of consecutive trials, one response type was rewarded on the majority of trials, controlled by two levels of probability (73% and 83%) known as feedback validity (FV), giving expected uncertainty in the environment. For a set of trials in which response type 1 was rewarded the most, we say that the underlying experimental rule was rule 1, likewise for type 2 responses and rule 2. Stimuli were presented in blocks of 120 trials having constant FV and one of two levels of volatility. High volatility blocks had a rule switch every 30 trials, stable blocks had the same underlying rule for all 120 trials. The participants were not given any information about the generation of rewards or the split of the task into blocks. This data has been examined previously, without considering the fit of different learning models to the behavior (Bland and Schaefer, 2011). Further details of the study can be found in the Materials and Methods section or Bland and Schaefer (2011).

**Figure 1:**
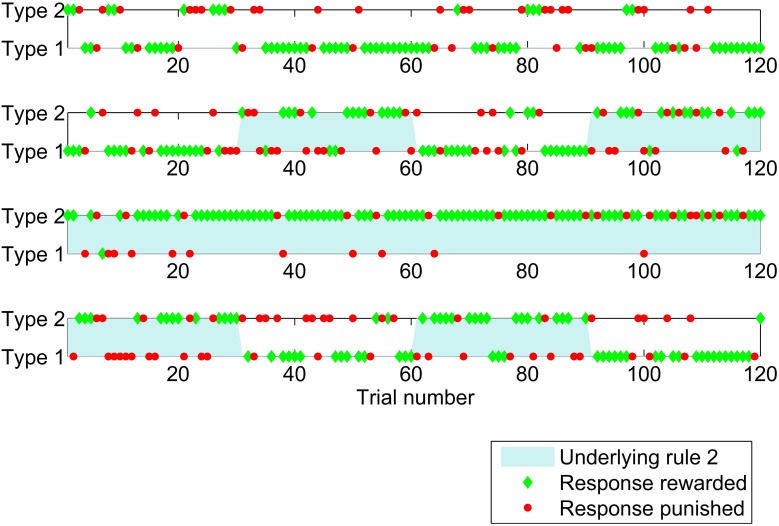
**Sequence of responses (a type 1 response is to press button one when a red triangle is shown and button two for blue, with type 2 the opposite) given by four participants for the first 120 trials of the study which had probabilistic feedback**. Responses which were positively rewarded (won points) are shown by green diamonds and responses which were penalized (lost points) are shown as red circles. The underlying experimental rule was rule 1 (responses of type 1 were rewarded the majority of the time) in the unshaded periods, and rule 2 in the shaded periods.

Figure 1 illustrates the study by showing responses made and feedback given for the first 120 trials of the study for four individual participants. Participants were most likely to switch from one response type to the other after negative feedback, that is a loss of points.

If the underlying rule can be identified from the pattern of rewards, but the result on individual trials cannot be predicted due to the randomness in reward generation, then to achieve the greatest rewards one should always respond according to the underlying rule, we call this *maximizing*. So if one knows that type 1 responses are rewarded mostly, then one should make a type 1 response every trial and ignore occasional losses. This does not consider how to identify which response is being rewarded most, or how to identify a rule switch. We quantify participants' behavior by calculating the percentage of trials in which each participant's response is of the type which is associated with the underlying experimental rule. Individual differences in responding to the task gave a range of maximizing behavior from 62% to 89% (mean 74.5%, *SD* 6%). The overall average feedback validity used in the experiment was 78%, so the average maximizing was just below that. This is in line with many other studies which find that in probabilistic tasks, the average frequency of each response matches the frequency of reward allocation, known as *probability matching* (see e.g., Vulkan, 2000; Shanks et al., 2002).

### 2.2. Computational modeling

Reinforcement learning models are based on standard reinforcement learning techniques of making trial by trial adjustments to the predicted value of a particular action, which is a prediction of how much reward is expected from that action. This predicted value is adjusted according to the outcome and a learning rate which controls how much influence the latest outcome has in changing the predicted value of an action. We considered three alternative reinforcement learning models, two of these, standard reinforcement learning model (RL) and win loss modified reinforcement learning model (WL), assume that the environment is fully coupled. This assumes that responses can be viewed in terms of the two response types described above, with the red triangle requiring the opposite response from blue, allowing us to ignore the actual color presented on each trial, and also that exactly one of the responses will be rewarded on each trial. This means that, if feedback shows that one response type is incorrect, then the other response type would have been correct on that trial and vice versa, so regardless of which response is made, feedback lets you know how each response type would have fared.

The assumption that the participants expected the environment to be coupled was motivated by the instructions given to participants, but to validate this assumption, we tested an uncoupled reinforcement learning model (UNC) which considers the colors seen and the button presses to be independent of each other and a separate predicted value is maintained for each combination of button and color. Given the assumption of independence, feedback after making a response does not give you any information about the result of pressing the other button or seeing the other color.

In our UNC and RL models, one learning rate is assumed for each participant. Our win loss modified reinforcement learning model (WL) allows wins and losses to have different influences on learning by allocating two learning rates to each participant, treating trials following a loss or a win separately.

Our Bayesian models are based on hidden Markov models which assume that rewards are governed by a hidden environmental state which cannot be directly observed but can be inferred. In our simple hidden Markov model (HMM), as with Hampton et al. (2006), the hidden state has two possible values, which are equivalent to the two experimental rules. Given the structure of the HMM and the outcomes, combination of response type made and feedback received, Bayesian reasoning is used to determine a probability of reward for each response type. Two sets of probabilities define the structure of the HMM, these are taken to be constant parameters for each participant. These probabilities control the chance of a rule switch and the relation between the hidden state and the reward.

Following the work of Behrens et al. (2007), we created a Bayesian model (VOL) which assumes an additional level of structure to the environment, volatility, or how quickly the environment is changing. As with Behrens et al. (2007), a hidden state relates directly to the probability of reward for a particular response, in our case representing the probability of response type 2 being rewarded, without the assumption in the HMM of only two states. This gives a flexible model which can respond to any change in state including changes in feedback validity. Like Behrens et al. (2007) we have assumed that the process for determining the current state and volatility does not vary between participants.

In all models, following the calculation of a belief or probability, we apply softmax action selection to determine the probability of making each action on each trial. Softmax action selection assumes that the chosen action depends on the difference between the values associated with each action and on a temperature parameter controlling the amount of randomization of responses on top of underlying beliefs. A low temperature increases the probability of choosing the higher valued action and a high temperature makes the probability of each action more similar. For the RL and UNC models we fit one temperature parameter to each participant's behavior, for the other models we fit two temperature parameters, differentiating trials following wins and losses.

Given a set of parameters and a model, we calculate a probability for each action on each trial for the outcomes received by the participant. The natural logarithms of the probabilities for the actions actually taken are summed for each participant. For each model, parameters are fit to each participant's behavior by searching possible values to maximize the likelihood of the parameters over all trials, for more details see Materials and Methods.

### 2.3. Comparing model fit

Models with more parameters should be able to show a closer fit to the data so it is customary to penalize models with more free parameters which have been fit to participants' behavior (Mars et al., 2012). To do this, we compare the five models described above by calculating the commonly used Bayesian Information Criterion (BIC) for each model (Lewandowsky and Farrell, 2011).

As a better model has a lower BIC value, Table 1 shows that the WL model gave the best overall fit to the data. We also examined the BIC for each model calculated separately for each participant. The UNC model was the worst fit to behavior compared to the other models for all participants. The best fit model was the WL model for 24 of the 30 participants, for four participants the best fit was the RL model and for two the HMM. Of the 24 participants for whom the WL model was the best fit, 23 had HMM as the next best fit. The differences in the BIC between the WL model and each other model were statistically significant, *p* < 0.001 in each case, *t*_(29)_ = 5.05, 4.25, and 7.48 for comparison of WL to RL, HMM, and VOL models, respectively.

**Table 1:** **Calculated BIC for all models using all participants**.

**Model**	**BIC**
RL	773.4
WL	726.3
HMM	742.8
VOL	851.1
UNC	998.0

**Figure 2:**
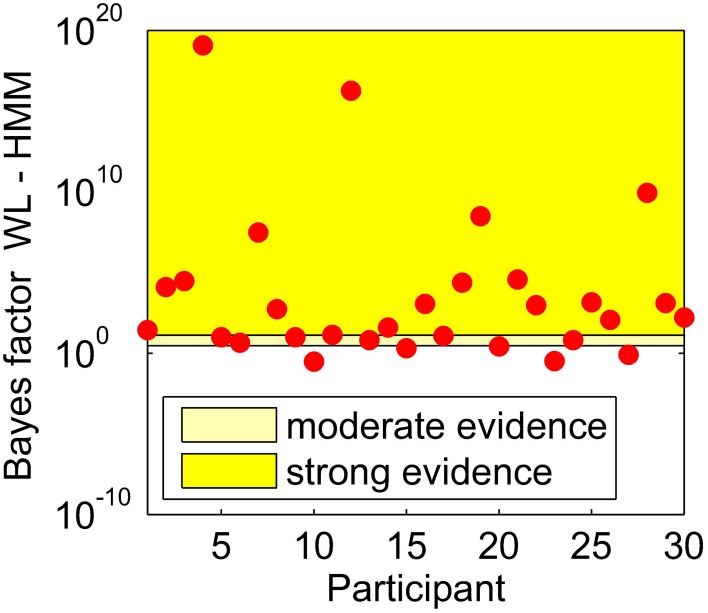
**Bayes factors for the difference between the WL and HMM models, calculated for each participant shown by a red circle**.

As described by Lewandowsky and Farrell (2011), we calculated Bayes factors for the difference between the HMM and WL models, we did this using the calculated BIC for each participant. Bayes factors can give an indication of the size of an effect, Lewandowsky and Farrell (2011) report previously proposed guidelines that a Bayes factor above 10 implies strong evidence for one model over the other, and between 3 and 10 implies moderate evidence. Figure 2 shows the Bayes factors for the WL compared to HMM for all participants.

**Figure 3:**
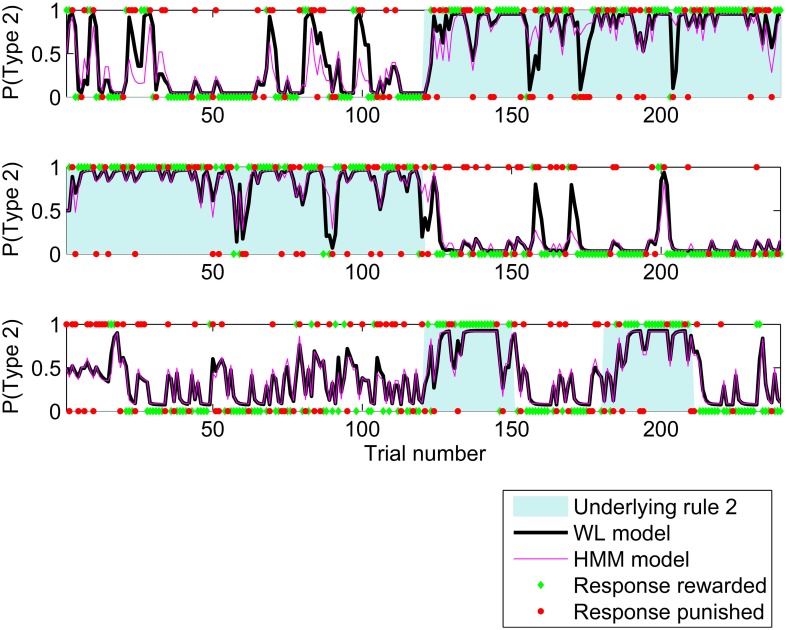
**Probability of a type 2 response calculated by HMM and WL models using the parameters fit to behavior by three participants for the first 240 trials of the study**. Unshaded areas indicate that, experimentally, rule 1 applied and shaded areas that rule 2 applied. The participants' responses are shown as 1 for type 2 responses, and 0 for type 1, responses which were rewarded are shown as green diamonds and those which were penalized as red circles.

The HMM and WL models fit the participants' behavior better than the other models so we now focus on these two models. Having used all trials to determine the best fit parameters for each participant and model, we could now calculate a trial by trial probability of making a type 2 response. Figure 3 shows these probabilities for the HMM and WL models for three participants for the first 240 trials of the study. In general, the probabilities match closely, but where there is a difference, the WL model is usually closer to the actual response made by the participant. This follows from the use of log likelihood to find the best model, Figure 3 is an illustration.

**Figure 4:**
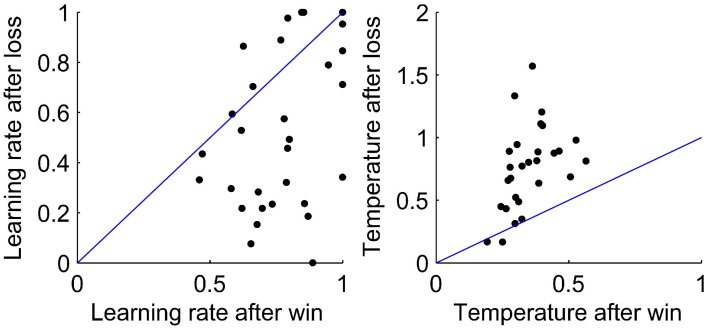
**Values of the fit parameters after a win and after a loss for each participant for the WL model**. **Left:** Learning rate. **Right:** Temperature. In each plot, a line is shown to indicate equality of the fit parameters after a win and after a loss.

As the WL model was the best fit to behavior, we look at the values of the fit parameters. Figure 4 shows the fit parameters for the WL model, the temperature was significantly higher after a loss than a win, *t*_(29)_ = 5.61, *p* < 0.0001 with means of 0.87 and 0.35 after a loss and a win, respectively. According to this model, participants were more likely to randomize their responses after a loss. The fit learning rates were significantly higher after a win than after a loss, means of 0.77 and 0.52, respectively, *t*_(29)_ = 4.52, *p* < 0.0001. A lower learning rate after a loss implies that losses are treated less strongly, which allows behavior to respond more slowly to occasional negative feedback and so take advantage of stable periods by not switching to the opposite response type when occasionally losing points when using the most likely response. Finally, we broke down the BIC scores for the WL model between a win and a loss. We find some indications that the WL model fits best after a loss, but this is at the edge of significance (*p* = 0.054).

### 2.4. Parameter recovery

If the fit parameters are reliable, we should be able to take simulated data, with known parameters, and accurately estimate those parameters (Lewandowsky and Farrell, 2011). For each model, we chose parameters to represent “typical participants” and used the model's learning rules to generate two sets of simulated responses to each participant's observed outcomes. We used the same process as for the original participant responses to estimate parameters for the simulated responses.

**Figure 5:**
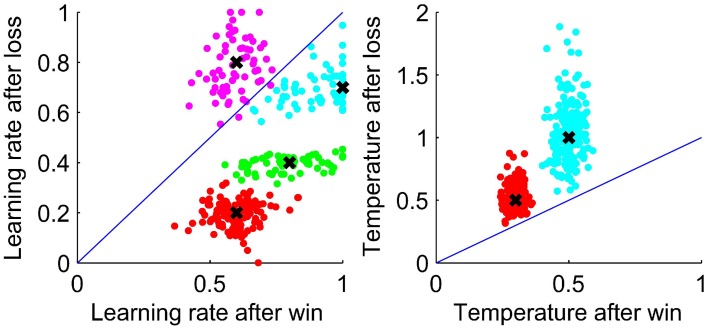
**Parameters estimated by the WL model for data simulated using the WL model with parameter values as shown by crosses**. **Left:** Learning rate. **Right:** Temperature. Lines show equality of the parameters after a win and after a loss.

Figure 5 shows that the fit parameters for the WL model are clustered around the parameters used for data generation which are shown by crosses suggesting that the parameters are reliable.

**Figure 6:**
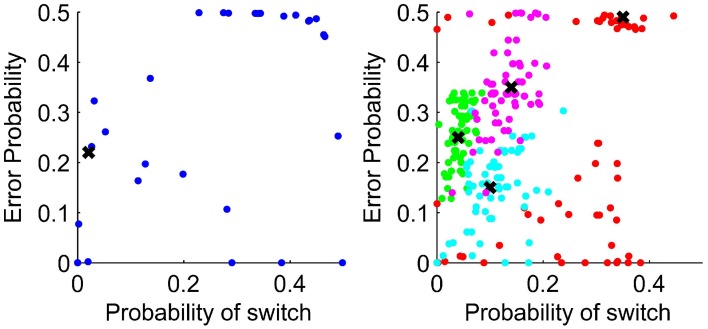
**Left:** Parameters fit to participant data for the HMM with the parameters to match the experimental generation of data shown by a cross. **Right:** Parameter values chosen to generate responses using the HMM are shown by crosses. Parameter estimates for the generated data are shown as dots. The probability of switch uses the assumption in the HMM that the environment switches from one in which rule 1 applies to rule 2 applies, and vice versa, with the same constant probability. The error probability is the probability of negative feedback when using the response type corresponding to the environmental rule which applies.

The left of Figure 6 shows the parameters representing probabilities in the structure of the HMM fit to participant behavior and on the right the parameters fit to data generated using the HMM with parameter values shown by crosses. If the participants had understood the experimental generation of outcomes and were applying that knowledge, we would expect the fit parameters to be close to those approximating the generation of data, shown in the left of Figure 6 by a cross.

For the HMM, the spread of fit parameters away from the data generation parameters shows that the parameters are not well recovered. In particular, for several participants the fit value for the error probability was 0.49, that is the probability of losing when using the response type associated with the current rule. Fitting parameters to data generated with this parameter value, the estimated parameter values covered the whole range of feasible values. For the data generated with parameters closer to the actual experimental data, the fit parameters are not so widely spread.

### 2.5. Model recovery

We found that the WL model was the best fit to participant data of the models tested. If model fitting is carried out on simulated data, the best fit model should be that which generated the data. Using the simulated data from the parameter recovery testing described above, we compared the fit of each model as in the analysis of participant data. Table 2 shows the percentage of simulations using each model which were best fit by each model. The correct model has been identified in most cases for all of the models.

**Table 2:** **Percentage of best fit models to simulations using each of the models**.

		**Simulated model**			
		**RL**	**WL**	**HMM**	**VOL**
**Fit model**	**RL**	99.6	8.6	18.8	0
	**WL**	0.4	86.0	1.6	0
	**HMM**	0	5.1	78.5	0.8
	**VOL**	0	0.3	1.1	99.2

The largest incorrect identification was the finding that the RL model was the best fit for 18.8% of the simulations by the HMM. The wrongly identified simulations were those which had the parameter for the error probability set to 0.49, and the probability of a switch set to 0.35. This was also the set of parameters which could not be reliably recovered from the simulated data as described above. A simulation using these parameters always gives probabilities close to 0.5 for each response with slight preference in line with the most recent outcome. Reinforcement learning produces responses in line with the most recent outcome by setting the learning rate to one, and the probabilities remain close to 0.5 by setting a high value for temperature. In this way the same behavior can be achieved by the HMM and RL models. Using BIC to compare models, RL will be preferred as the RL model has two parameters compared to four for the HMM.

### 2.6. How well can these learning methods do?

Human behavior was best fit by the WL model, we now consider how the models compare when carried out by an ideal agent. By ideal agent, we mean an agent which always selects the action which the model suggests is most likely to give a reward, and the model parameters are chosen to give the highest number of rewards for the task. We used the sequence of outcomes received by each participant in the task and then considered the performance of each model on each participant's trials.

For the RL and WL models, these parameters were found by a grid search over all possible values of the learning rates at intervals of 0.01. For the WL model, a learning rate after a win of 0.48 and after a loss of 0.24 maximized rewards. A learning rate of 0.2 gave maximum rewards for the RL model. The WL model won significantly more rewards than the RL model *t*_(30)_ = 3.53, *p* = 0.0014.

For the HMM, we searched the parameter space in the region of those parameters approximating the generative environment to find the best performance. The generative environment had equal blocks with FV of 83% and 73%, giving an average probability of 22% of losing when using the response associated with the rule, the error probability. For the probability of a rule switch we used a probability of 0.021 based on 5 switches in 240 trials, having switches after 120 or 30 trials. The parameters which maximized rewards were 0.021 for the switch probability and 0.2 for the error probability. There was no significant difference between the performance of the WL and HMM models *t*_(30)_ = 1, *p* = 0.33. The HMM was significantly better than the VOL model, *t*_(30)_ = 4.98, *p* < 0.0001.

**Figure 7:**
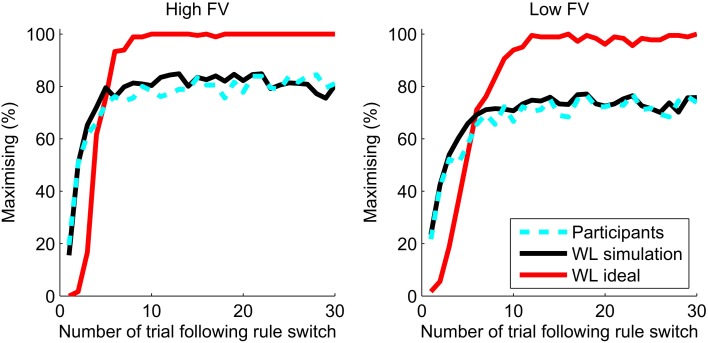
**Maximizing after a switch for the best parameters for the WL model (WL ideal, red line) compared to the percentage of maximizing responses by participants (dashed cyan line)**. The WL simulation uses parameters fit to each participant's behavior.

Figure 7 shows the maximizing behavior, aligned with the experimental rule, of the ideal WL model in comparison to that of the participants. The percentage of responses in line with the underlying experimental rule were averaged over all participants and the ideal WL model for trials following rule switches, with each of the levels of feedback validity (FV) shown separately. The ideal WL has parameter values which optimize behavior over all trials, not just volatile blocks. The ideal WL model far outperforms the participants and reaches a steady level of maximizing at 100% in the high FV condition.

As well as being able to outperform humans, the WL model can also closely simulate human behavior. Ten sets of simulated responses were generated using the WL models with the fit parameters and the sequence of outcomes for each individual participant. Figure 7 shows that the simulations closely replicate the aggregate performance of the participants. Although only volatile blocks are shown, the parameters used in the simulations were those fit to participant behavior across all trials regardless of the experimental conditions. Maximizing behavior of participants and simulations quickly adapts to a rule switch and reaches a plateau which is approximately equal to the level of feedback validity (probability matching). For trials 21–30 following a switch in the high feedback validity (FV = 83%) condition, participants showed maximizing of 82% and the WL simulation 81%. In the low feedback validity condition (FV = 73%), maximizing by participants and the WL model was 73%.

## 3. Discussion

We find that a reinforcement learning model with separate parameters after a win or a loss (WL) gives a significantly better description of human behavior in a two-alternative probabilistic learning task with rule reversals than the other models tested. Our VOL model, implementing the model of Behrens et al. (2007), is flexible and able to adapt to changes in the level of expected uncertainty, or feedback validity (FV), and volatility. However, the WL model is a better fit than the others although it has constant parameters throughout all trials. Behrens et al. (2007), in a broadly similar decision making task, found their model to be a better fit to human behavior than reinforcement learning with constant parameters for each participant or separate parameters for volatile and stable blocks. The difference between the fit of our WL model and standard reinforcement learning (RL) applies even when they are compared using a method that penalizes the WL model. In particular, the advantage of the WL model was observed when the BIC of the models was compared, a method which strongly penalizes models with higher numbers of parameters such as our WL model (Lewandowsky and Farrell, 2011).

Comparing the performance of ideal agents on our task, we find no significant difference between the HMM and WL models. Ideal agents have parameters which are chosen to maximize rewards given the model and always choose the option given by the model as the most favorable. Bayesian models are constructed to make optimal decisions, providing that the assumptions underlying the models are correct. Although the assumptions of the Bayesian models are based on the experimental structure used to generate rewards, the HMM or VOL models do not outperform the WL model on our task although the WL model does not adjust its learning rate to accommodate different levels of unexpected uncertainty and volatility. There is a small but significant improvement in performance of the WL model compared to the RL model when using ideal agents. All of the models, when used by ideal agents, far outperform human behavior.

In our WL model, we find that for both the learning rate and temperature parameters, there is a significant difference between the fit parameter values following a win and a loss. These differences are consistent with psychological studies reporting behavioral differences in response to wins and losses and with existing neuroscientific knowledge indicating the existence of different neural pathways linked to the processing of wins and losses (see e.g., Kravitz et al., 2012; Yechiam and Hochman, 2013b).

In psychology, the concept of loss-aversion, which suggests that behavior changes more in response to losses than to gains of similar magnitude (Kahneman and Tversky, 1984) has prompted much investigation. As an alternative mechanism to loss-aversion, Yechiam and Hochman (2013b) proposed a loss-attention mechanism in which losses cause participants to attend more closely to a task and so losses decrease the amount of randomization.

These ideas of loss-aversion are often tested in studies of response to risk, that is where participants choose between alternatives with known outcome probabilities. An example (from Kahneman and Tversky, 1984) is a choice between a safe or risky option, where the risky option has an 85% chance of winning $1000 and a 15% chance of winning nothing and the safe option pays out $800 with certainty. People tend to prefer the safe option. In their examination of the loss-attention hypothesis, Yechiam and Hochman (2013a), used several tasks which involved repeated selections between a safe and a risky option where the probabilities had to be learnt from experience. They tested their loss-attention model by fitting a choice sensitivity parameter, the inverse of our temperature parameter, for each task. They found less randomization of responses in tasks in which losses were possible compared to tasks without losses. In our task, unlike that of Yechiam and Hochman (2013a), the participants could not avoid losses as there was no way to predict the outcome on individual trials. We find a higher temperature after individual losses, implying that participants are less likely to follow the underlying belief after a loss. This does not necessarily conflict with the idea of loss-attention, as adding randomness to a response after a loss may be a mechanism for testing an underlying belief without making a large adjustment to that belief.

In neuroscience, dopamine is related to reward and punishment and separate D1 and D2 dopamine receptors in the basal ganglia have been found to respond to reward and punishment, respectively (see e.g., Gerfen, 1992). This inspired the use of separate pathways to respond to reward and punishment in the computational neural models of reinforcement learning by Frank and colleagues (see e.g., Frank, 2005; Samson et al., 2010). Testing this, Kravitz et al. (2012) found different pathways in the striatum of mice to be involved in processing reward and punishments. Rather than indicating reward and punishment directly, Schultz and colleagues suggested that dopamine signals the difference between an expected reward and that actually received (see e.g., Schultz, 1998). This difference forms the prediction error which is calculated in reinforcement learning.

Following their neural models with separate pathways for learning after a win and a loss, (see e.g., Frank, 2005; Samson et al., 2010). Frank et al. (2007) use separate learning rate parameters following a win and a loss when using a reinforcement learning model to analyze human behavior in a probabilistic task. Like us, they find that the mean learning rate following a win is higher than that after a loss. They use just one temperature parameter and, as they are looking at associations between genetics and reinforcement learning parameters, they do not compare alternative models of behavior.

We are aware of only a few studies that have considered separate effects of reward and punishment when comparing alternative computational models of learning from experience, none of which compare Bayesian models which make assumptions about the nature of the environment. These studies are based on different learning tasks to ours, and fit different reward values following a win or a loss (e.g., Ito and Doya, 2009; Guitart-Masip et al., 2012). Guitart-Masip et al. (2012) had four fractal images which signalled whether participants should respond or not to gain rewards or avoid punishments, these associations had to be learnt from experience and there was no switch in associations. Guitart-Masip et al. (2012) fit a number of different reinforcement learning models to behavior, the best fit model did not scale rewards and punishments differently. Analyzing the decisions of rats in two-stage probabilistic decisions, Ito and Doya (2009) found that a reinforcement learning model with different reward values after a win and a loss was a better fit to the rats' behavior than reinforcement learning without differentiation between wins and losses. To maintain the symmetry of the task in which exactly one response is correct on each trial, we have taken a different approach and fit a separate learning rate, rather than reward value, following wins and losses.

As our Bayesian and reinforcement learning based models make different assumptions about the environment, comparing the fit of different models to human behavior can give insights into the assumptions people make about the environment. Our HMM, as with that of Hampton et al. (2006), as well as assuming that the two outcomes are coupled, assumes that there will be rule switches within probabilistic feedback. Our VOL model not only assumes that there will be rule switches, but also that the frequency of switches depends on the level of volatility in the environment. Hampton et al. (2006) compared a hidden Markov model to a reinforcement learning model that made no assumptions about the structure of the environment. They concluded that participants make assumptions about the structure of the environment. We also found that our hidden Markov model (HMM) was a better fit to behavior than a reinforcement learning model which did not assume that the outcomes were coupled. This uncoupled reinforcement learning model, however, was not as good a fit as our RL and WL models. From this we conclude that participants made some assumptions about the environment but have no evidence that they adjusted their rate of learning to the structure of the environment.

Bayesian models can optimize the number of rewards received when the assumed structure for the Bayesian inference exactly matches the underlying structure of the task. We examined the performance of our models when, rather than being fit to human behavior, the model parameters were selected to maximize the total reward achieved, we refer to this as an ideal agent using the model. In our task, the rewards obtained by an ideal agent using the WL model were not significantly different to those of the ideal HMM. Our ideal HMM has parameters to closely resemble the generative structure, but assumes that there is a small constant probability of a rule switch. In the experimental data, rule switches only occurred at the ends of blocks of 30 or 120 trials. The HMM also assumes that for each environmental state, there is a constant probability of each outcome. However, the experimental data was generated using two levels of FV, with outcomes randomized to give the correct proportion within a block. We do not believe that these differences between the generative process and the assumptions of the HMM significantly hamper the performance of the HMM. We believe that the ideal agent using the WL model is approaching an optimal level of response in this task. The ideal WL model had a small but significant advantage over the ideal RL model in our task. Our ideal HMM also performed significantly better than our VOL model, our implementation of the model of Behrens et al. (2007). The VOL model is more flexible in the situations in which it can learn.

The parameters for the ideal WL model, those which gave the best performance in the task, were learning rates of 0.48 after a win and 0.24 after a loss. Our participants also had significantly higher learning rates after a win than a loss, although generally higher than the ideal parameters, with means of 0.76 and 0.52 after a win and a loss, respectively.

Learning under expected uncertainty with volatility is not simple as indicated by the range of participants' responses. However, our task, having coupled outcomes in which one or the other response is correct, does not require any exploration, or trying the different alternatives to see if things have changed. We expected the participants to know that if the button press was incorrect, then the other button would have been correct. Exploration is an important feature of learning from experience (see e.g., Cohen et al., 2007). Tasks which have more than two options automatically require exploration, as negative feedback does not show what would have been the correct response. It will accordingly be more difficult to learn when there are more alternatives. It has been acknowledged that standard reinforcement algorithms are not suitable in complex situations in which there may be many possible states or actions (e.g., Botvinick et al., 2009). Wilson and Niv (2012) compared optimal performance between a Bayesian and non-Bayesian model in their probabilistic learning task and the Bayesian model clearly had superior performance. Our finding that our ideal WL reinforcement learning model performs as well as our HMM may be restricted to the case of coupled two alternative tasks. Additionally, the level of feedback validity might affect the relative performance of the different styles of responding. These issues remain to be investigated.

We assumed that whatever decision making processes the participants use to make their responses, these remain constant for the whole task. We have assumed that the task instructions give participants enough information to form a model. Some studies have found that a Bayesian model is a better fit to human behavior only in conditions when participants have been told to expect changes in rule (Payzan-LeNestour and Bossaerts, 2011; Wilson and Niv, 2012). In our study, participants were not given such information. The instructions given to participants can affect behavior in other ways, Taylor et al. (2012) found that probability matching was influenced by whether participants had an explanation for the probabilistic outcomes.

In summary, we conclude that, with distinctions between learning from a win and a loss, reinforcement learning provides a very good description of the participant responses to repeated trials under expected uncertainty and volatility. It is able to account for individual differences with parameters that remain constant throughout all trials although the feedback validity and volatility varied. Future research should explore whether the differential treatment of a win and a loss would lead to a similar robust performance in other experimental situations.

## 4. Materials and methods

### 4.1. Behavioral measurements

Here we give a brief outline of the methods used for recording the behavioral data, for full details see Bland and Schaefer (2011). The sample of this study was formed by thirty-one participants (mean age = 24, *SD* = 4.99), 18 of whom were female. The study was approved by the local Ethics committee and participants gave written informed consent. For this computational analysis, we have excluded one participant whose behavioral performance showed maximizing at less than 55%. Participants were told that they started with 1000 points and would win or lose points according to their responses and that they would earn a payment based on their final points total. On each trial, participants were shown a colored triangle and had to respond to this stimulus by pressing one of two buttons. They were instructed, “This triangle will be either blue or red and your key press should be a guess about which is the right answer in response to the triangle. You will learn which is the correct answer.” There are no explicit instructions on whether the responses were coupled or uncoupled. However, the structure of the task is such that accurate performance is not attainable without learning that the responses are complementary. The levels of performance that we observed are a demonstration that participants were not assuming that responses were uncoupled (and a demonstration that probably most of them were learning very quickly that the responses were coupled).

Participants were given immediate on screen feedback as to whether they were correct and had won 10 points, were wrong and lost 10 points or were too slow to respond (over 1500 ms) and also lost 10 points. Participants were not told about underlying rules regarding rewards, or given any indication of changes in experimental condition, and were not given a running total of points. They were asked to try to win as many points as possible.

The environment could be considered to have a current underlying rule which was manipulated by the experimenters. Rule 1 meant that responses of type 1 were rewarded on the majority of trials, where type 1 responses were to press button one when shown the red triangle and button two for blue, so rule 1 is an association between red and button one. Similarly, rule 2 meant that responses of type 2 were rewarded on the majority of trials, where type 2 responses were to press button one when shown a blue triangle and button two for red. Responses were rewarded at two different probabilities or levels of feedback validity (FV) which remained constant through blocks of 120 trials. In high FV blocks, responses in line with the current rule were rewarded on 83.3% of trials, and in low FV blocks this was 73.3% with the actual outcome on individual trials randomized to meet these percentages. Two levels of volatility for a block gave two different frequencies of switches, in stable blocks, the environmental rule was constant for all 120 trials. In volatile blocks, the rule switched every 30 trials. Having two rules, two levels of FV and two levels of volatility gave eight conditions which were presented in blocks of 120 trials. All participants experienced all conditions but in a randomized order.

### 4.2. Updating beliefs

#### 4.2.1. Reinforcement learning models

***4.2.1.1. Uncoupled Reinforcement Learning (UNC)***. Reinforcement learning considers the predicted value of, or rewards which will be obtained by taking a particular action. In our uncoupled reinforcement learning, four separate action values are maintained, for each combination of color seen and button pressed. The reward value is taken to be 1 for a win and 0 for a loss. At each trial a prediction error, δ(*t*), is calculated as the difference between the reward and the predicted value of the response made to the color shown as follows

$$\delta (t)=R(t)-Q_{i}(t),$$

where *Q*_*i*_(*t*) represents the predicted value of the response made to the color shown. This prediction error, δ(*t*), is used to update the expected value *Q*_*i*_(*t*) for the next trial, using a learning rate, α, with a value between 0 and 1 as follows

$$Q_{i}(t+1)=Q_{i}(t)+\alpha \delta (t).$$

All *Q*_*j*_(*t*) for color and button combinations not experienced on that trial are maintained for the next trial, without any forgetting. Each participant was considered to have one constant value for α regardless of the color and button combination being updated.

***4.2.1.2. Standard reinforcement learning (RL)***. In this form of reinforcement learning, we consider the colors and buttons to be opposites, and use the response types as described above, in this formulation, every trial gives full information about each possible action and so only one predicted value needs to be maintained. Suppose *Q*_1_(*t*) is the predicted value of using response type 1, on trial *t* and that *R*(*t*) is the reward associated with response type 1. We encode the reward, *R*, according to whether the feedback was a win or a loss, ignoring the actual number of points won and lost, by setting *R* to 1 if a response of type 1 was rewarded on that trial and set to 0 otherwise. This meant that *Q*_1_(*t*) could take values between 0 and 1, as the maximum reward which could be expected on a trial was 1. As we assume that the situation was coupled and each trial can result in a win or a loss, on trials when response type 2 is carried out, we take the opposite feedback to be that related to response type 1. At each trial a prediction error, δ(*t*), is calculated as the difference between the reward and the predicted value of a type 1 response as follows

$$\delta (t)=R(t)-Q_{1}(t).$$

This prediction error, δ(*t*), is used to update the expected value of a type 1 response for the next trial, using a learning rate, α, with a value between 0 and 1 as follows

$$Q_{1}(t+1)=Q_{1}(t)+\alpha \delta (t).$$

The learning rate determines how quickly new information is incorporated into beliefs. A learning rate of 1 results in only the most recent trial being taken into account. As there is no reason to expect one response type to be better than the other at the start of the trials, the initial value for *Q*_1_(*t*) is set to 0.5. As *Q*_1_(*t*) is always between 0 and 1 and the two response types are opposites, the expected value for a type 2 response is calculated as *Q*_2_(*t*) = 1 − *Q*_1_(*t*).

In our standard reinforcement learning (RL) model, the learning rate, α, is considered to be a constant for each individual and was determined by parameter fitting as described below.

***4.2.1.3. Win loss modified reinforcement learning (WL)***. In this model, the predicted values for responding in accordance with each rule are calculated exactly as for standard reinforcement learning but in the WL model, each participant is assumed to have two different learning rates which apply according to whether they received a reward or punishment on the previous trial.

#### 4.2.2. Bayesian models

***4.2.2.1. Hidden markov model***. To derive the calculations used in the Bayesian models, we use random variables to denote aspects of the task. A random variable is one which can exist in one of a finite number of mutually exclusive states. The outcome on trial *t* in the study is represented by the random variable *Y*_*t*_ and has two possible values for the two response types which can be rewarded. We use the notation **P** to denote a set of probabilities, so **P**(*Y*_*t*_) represents a probability for each value which *Y* can take. These sets of probabilities can be represented as column vectors, so for example for *Y*, $\left(\begin{array}{c} a\\ b \end{array}\right)$ can indicate that there is a probability *a* that a type 1 response is rewarded and *b* that type 2 is rewarded. We use *y*_*t*_ as shorthand for *Y*_*t*_ = *y* where *y* is the known (but arbitrary) value taken by *Y* on trial *t*. When an outcome has been observed, then the probability becomes 1 for one response type and 0 for the other, so *y*_*t*_ can be represented by $\left(\begin{array}{c} 1\\ 0 \end{array}\right)$ or $\left(\begin{array}{c} 0\\ 1 \end{array}\right)$.

The hidden Markov model (HMM) used in this work is broadly based on the work of Hampton et al. (2006). This model assumes that the outcome on each trial depends only on the value of a hidden state at that trial. At trial *t* the hidden state is represented by *X*_*t*_ and has two possible values, denoted by *x*^*i*^ where *i* can be 0 or 1. In each state one of the two response types is rewarded the majority of the time. In this model, the hidden state only depends on its value at the previous trial and on the set of constant probabilities, **P**(*X*_*t*_|*X*_*t*−1_), for staying in the same state or switching. Given a hidden state, there is assumed to be a constant probability for each possible outcome *y*, this set of probabilities is written **P**(*Y*_*t*_|*X*_*t*_). In matrix notation the form of both **P**(*X*_*t*_|*X*_*t*−1_) and **P**(*y*_*t*_|*X*_*t*_) is given by:

$$\left(\begin{array}{cc} p & 1-p\\ 1-p & p \end{array}\right)$$

where *p* is between 0 and 1 and the values for *p* in **P**(*X*_*t*_|*X*_*t*−1_) and **P**(*y*_*t*_|*X*_*t*_) are different. Representing the parameters this way assumes that there is symmetry in the underlying environment. This representation assumes that the probability of a switch from one environmental state to the other is the same whichever of the two states the environment is in initially. It also assumes that if one response type is rewarded with a set probability in one environmental state then the other response is rewarded with the same probability in the other state. The probabilities are considered to be parameters which are fit to the behavior of the participants.

The HMM assumes that the participants estimate these two sets of probabilities and that they do so quickly enough that they can be considered to be constants. It also assumes that it is not necessary to estimate the two different experimental levels of feedback validity and that participants do not realize that switches only occur at 30 trial intervals.

**Figure 8:**
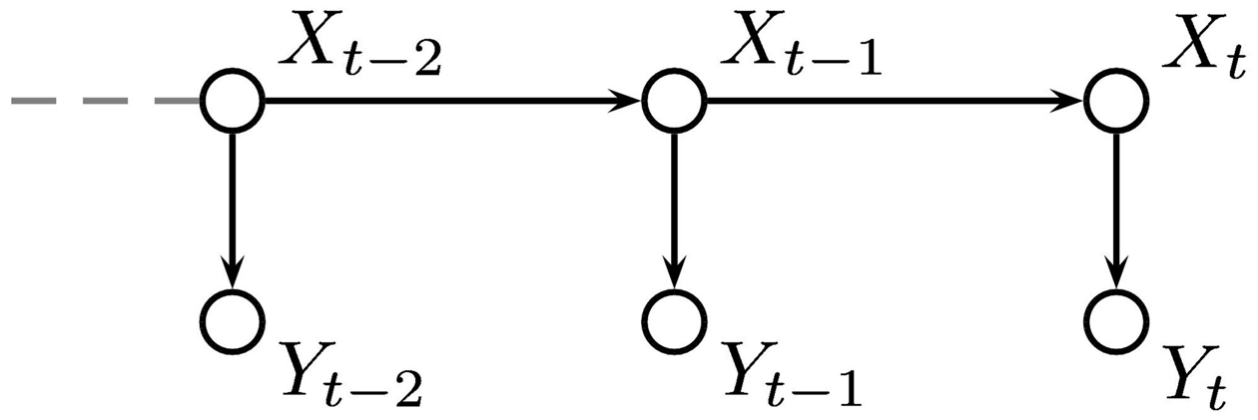
**Graphical representation of our hidden Markov model (HMM)**. The environmental state at trial *t* is denoted by *X*_*t*_ and its value depends only on its value at the previous trial. The outcome is represented by *Y*_*t*_ and depends only on *X*_*t*_.

If we have values, or estimates, for the probabilities of the environment being in each possible state after *t* trials, **P**(*X*_*t*_, *y*_1_, …, *y*_*t*_), then we can incorporate the probability of a switch in state **P**(*X*_*t*_|*X*_*t*−1_) to give an estimate for the probabilities for each state at trial *t* + 1 which can be used to inform our responses. When an outcome is observed, the probabilities can be updated using the probabilities of the outcome actually observed given each hidden state, **P**(*y*_*t*_|*X*_*t*_). This gives a process which can be used at each time step and only requires the probability distribution for *X* to be stored. Figure 8 shows the relationship between the variables in the HMM.

To show this process formally, we take the joint probability distribution at trial *t* for *X* with all the known observations *y*_1_, …, *y*_*t*_, written **P**(*X*_*t*_, *y*_1_, …, *y*_*t*−1_, *y*_*t*_), and use the definition of conditional probability to write

(1) $$P(X_{t},\;y_{1},\;\ldots ,\;y_{t-1},\;y_{t})=P(y_{t}|X_{t},\;y_{1},\;\ldots ,\;y_{t-1})P(X_{t},\;y_{1},\;\ldots ,\;y_{t-1}).$$

But *y*_*t*_ depends only on *X*_*t*_ so

(2) $$P(y_{t}|X_{t},\;y_{1},\;\ldots ,\;y_{t-1})=P(y_{t}|X_{t}).$$

Substituting Equation (2) into (1) gives

(3) $$P(X_{t},\;y_{1},\;\ldots ,\;y_{t-1},\;y_{t})=P(y_{t}|X_{t})P(X_{t},\;y_{1},\;\ldots ,\;y_{t-1}).$$

Now we introduce variable *X*_*t*−1_ because we know that

(4) $$P(X_{t},\;y_{1},\;\ldots ,\;y_{t-1})=\sum_{i}P(X_{t},\;x_{t-1}^{i},\;y_{1},\;\ldots ,\;y_{t-1})$$

where *i* takes values 0 and 1. Using the definition of conditional probability, Equation (4) can be re-written to give

(5) $$P(X_{t},\;y_{1},\;\ldots ,\;y_{t-1})=\sum_{i}P(X_{t}|x_{t-1}^{i})P(x_{t-1}^{i},\;y_{1},\;\ldots ,\;y_{t-1}).$$

Now Equation (5) can be substituted in to Equation (3) to give

(6) $$P(X_{t},\;y_{1},\;\ldots ,\;y_{t})=P(y_{t}|X_{t})\sum_{i}P(X_{t}|x_{t-1}^{i})P(x_{t-1}^{i},\;y_{1},\;\ldots ,\;y_{t-1}).$$

The left hand side in Equation (6) is now written in terms of its value on the previous trial when combined with known probabilities. To begin the process, it is assumed that there is an equal probability of *X* being in either of the two possible states.

***4.2.2.2. Hidden markov model with volatility***. The work of Behrens et al. (2007) led to our hidden Markov model with volatility (VOL). Being based on a hidden Markov model, this model shares some features with the HMM described above but makes different assumptions about the nature of the environment.

In the VOL model, as in the HMM, the outcome at a particular trial depends only on the value of a hidden state at that trial. In this case the hidden state, *X* represents the probability that a type 2 response will be rewarded. As *X* represents a probability, it must have values between 0 and 1. For computation, *X* was treated as a discrete random variable by taking 49 equally distributed points in the 0, 1 interval. Responses can be based on the mean, or expected value, of the probability distribution over *X*, that is the probability that *X* takes each of its possible values.

**Figure 9:**
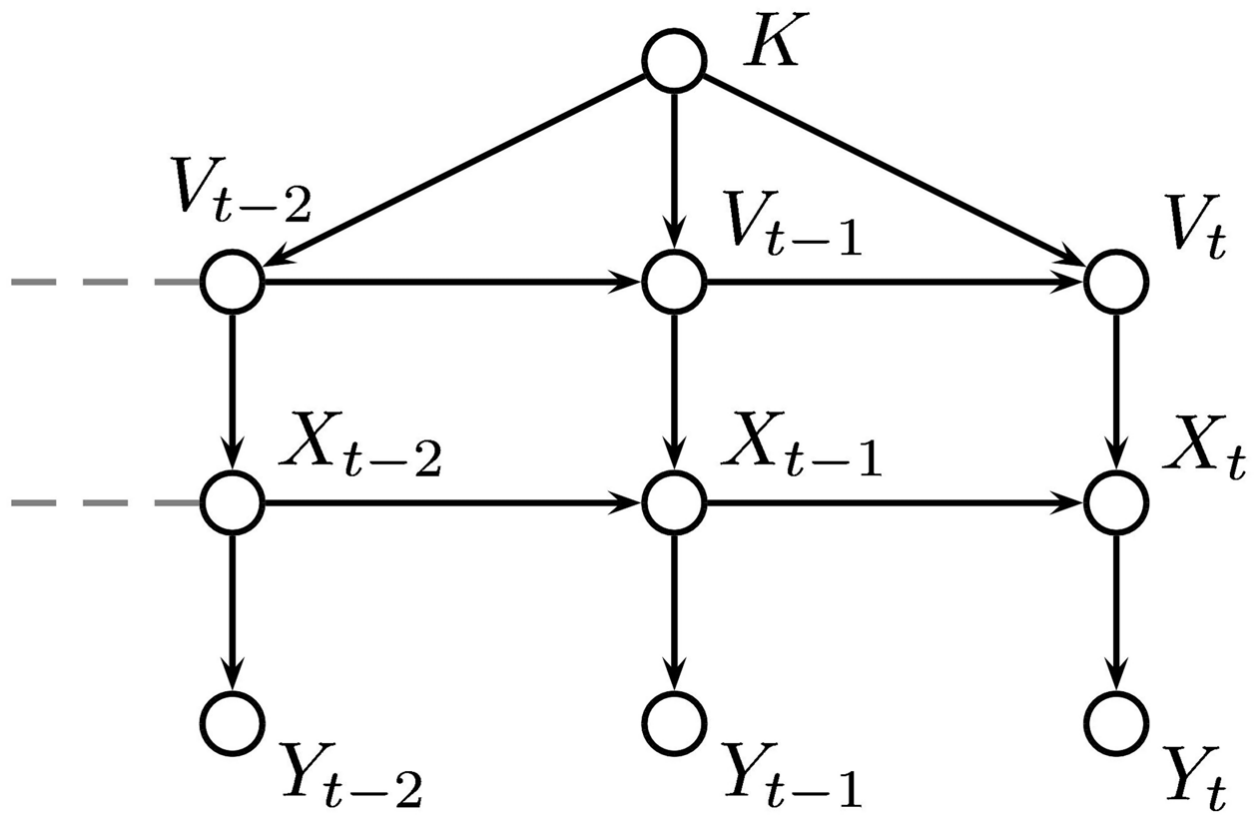
**Graphical representation of our VOL model based on the work of Behrens et al. (2007)**. The variable, *X* represents the probability of a type 2 response being rewarded and the volatility, *V* determines the probability of *X* taking a value unrelated to its current one. The parameter, *K* is a measure of trust in the estimated value of the volatility.

As in the HMM, the value of *X* depends on its previous value, but in the VOL model, *X* also depends on the value of a second hidden variable, *V*, representing the volatility of the environment, which was also treated as a discrete random variable taking values between 0 and 1 in the same way as *X*. The volatility, *V*, depends on its previous value and that of a parameter *K*. The parameter *K* is a representation of the degree of confidence in the estimate for volatility. The relationships between the variables in the VOL model are shown in Figure 9.

The equations for updating probabilities for the VOL model follow in a similar way to those for the HMM. For transitions from one trial to the next, there are sets of probabilities **P**(*V*_*t*_|*V*_*t*−1_, *K*) and **P**(*X*_*t*_|*X*_*t*−1_, *V*_*t*_) for transitions of *V*_*t*_ and *X*_*t*_, respectively. In this case the joint probability to consider is **P**(*X*_*t*_, *V*_*t*_, *K*, *y*_1_, …, *y*_*t*_) and the outcome can be incorporated as follows

(7) $$P\left(X_{t},\;V_{t},\;K,\;y_{1},\;\ldots ,\;y_{t}\right)=P\left(y_{t}|X_{t}\right)P\left(X_{t},\;V_{t},\;K,\;y_{1},\;\ldots ,\;y_{t-1}\right).$$

Now we can introduce and sum out over *X*_*t*−1_ as before to give

(8) $$P\left(X_{t},\;V_{t},\;K,\;y_{1},\;\ldots ,\;y_{t}\right)=P\left(y_{t}|X_{t}\right)\sum_{i}P\left(X_{t}|x_{t-1}^{i},\;V_{t}\right)P\left(x_{t-1}^{i},\;V_{t},\;K,\;y_{1},\;\ldots ,\;y_{t-1}\right).$$

Now we need to also sum out over *V*_*t*−1_ in a similar way, giving

(9) $$P\left(X_{t},\;V_{t},\;K,\;y_{1},\;\ldots ,\;y_{t}\right)=P(y_{t}|X_{t})\sum_{i}P(X_{t}|x_{t-1}^{i},\;V_{t})\sum_{j}P\left(V_{t}|v_{t-1}^{\;j},\;K\right)P\left(x_{t-1}^{i},\;v_{t-1}^{\;j},\;K,\;y_{1},\;\ldots ,\;y_{t-1}\right).$$

Equation (9) gives an expression for **P**(*X*_*t*_, *V*_*t*_, *K*, *y*_1_, …, *y*_*t*_) in terms of its value on the previous trial.

Following the ideas of Behrens et al. (2007), we used a beta distribution, with a mean of the old value of *X*, to determine the probability distribution for *X* at the next time step. Also motivated by Behrens et al. (2007), we used a normal distribution to determine the probability distribution for *Y*. The actual distributions used for transition matrices and initial distributions were based on our previous investigation (Duffin, 2011) into replicating the behavior of the model of Behrens et al. (2007).

### 4.3. Probabilities for actions

The learning models give a predicted value or probability for making a particular response at each trial, this can be considered to be a belief at trial *t*, *B*(*t*). For the UNC model, the belief is a value for making each button press, given the color that is displayed, for the other models, the belief is based on making a type 1 response. People do not always respond in accordance with the underlying belief, so a softmax rule is often used to select the action (Daw et al., 2006). A softmax rule varies the amount of randomization of responses according to the difference between the beliefs in the two options. A temperature parameter, *T*, is used to control how much randomization is used. A low temperature gives a high probability of choosing the action with the highest belief, even when the beliefs are quite close. A high temperature results in mainly random responses, giving probabilities close to 0.5 for each of the actions, for the models which have only two actions. Using the softmax rule and given a belief *B*(*t*) in a type 1 response, the probability, *P*(*t*), of making a type 1 response is given by

$$P(t)=\frac{e^{\frac{B(t)}{T}}}{e^{\frac{B(t)}{T}}+e^{\frac{1-B(t)}{T}}}.$$

As there were only two possible actions, the probability of making a type 2 response was given by 1 − *P*(*t*).

For each of the models apart from the uncoupled and standard reinforcement learning models (UNC and RL), each participant was assumed to have two different temperature parameters, for after positive or negative feedback. When a participant failed to respond in the allowed time, we assumed that the previous belief value would be remembered and that the temperature parameter applied would be that used after a loss.

### 4.4. Fitting parameters

For each model, a set of suitable parameters for that model and a sequence of outcomes observed by an individual participant, the process described above can be used to calculate a probability for each response type at each trial. The full joint probability of the data given a set of parameters for a participant is given by the product of the probability of each response actually made. This assumes that given an underlying model, each response is independent of each other. The likelihood of a set of parameters given data is defined to be the probability of the data given parameters. For each participant we took the sum of the log likelihood for each response actually taken to give the total log likelihood of the parameters. Trials in which a participant failed to respond were not included in the log likelihood calculations. We found parameters to maximize the likelihood, for each participant and model, by using the search function fmincon in Matlab to minimize the negative of the log likelihood. Parameters were constrained according to the model. For each model we allowed temperature parameters to take any value greater than or equal to 0.01. For the reinforcement learning models, we allowed the learning rate to take values between 0.0001 and 1 inclusive. For the HMM, the probability parameters took values between 0.00001 and 0.5 inclusive.

The parameter fitting process was done for each participant and model and gave a set of best fit parameters and a log likelihood value for those parameters for that participant and model.

### 4.5. Comparing models

Bayesian Information Criterion (BIC) is used to take into account the number of parameters and is given by

(10) BIC=−2 logL+k logN

where *L* is the likelihood, *k* is the number of parameters in the model, *N* is the number of data points and natural logarithms are used. Lower BIC values imply a better fit to the data.

### Conflict of interest statement

The authors declare that the research was conducted in the absence of any commercial or financial relationships that could be construed as a potential conflict of interest.
